# Sarcopenia Is Associated with Altered Rocuronium Onset and Neuromuscular Blockade Kinetics in Liver Transplant Recipients: A Prospective Observational Cohort Study

**DOI:** 10.3390/jcm15103620

**Published:** 2026-05-08

**Authors:** Emre Arikan, Neslihan Altunkaya Yagci, Sami Akbulut, Yusuf Ziya Colak, Duygu Demiroz, Ahmet Kadir Arslan, Nurullah Dag, Feti Ahmet Engin, Nurcin Gulhas, Muharrem Ucar

**Affiliations:** 1 Department of Anesthesiology and Reanimation, Inonu University Faculty of Medicine, 44280 Malatya, Türkiye; 2 Department of Surgery and Liver Transplantation, Inonu University Faculty of Medicine, 44280 Malatya, Türkiye; 3 Department of Biostatistics and Medical Informatics, Inonu University Faculty of Medicine, 44280 Malatya, Türkiye; 4 Department of Radiology, Inonu University Faculty of Medicine, 44280 Malatya, Türkiye

**Keywords:** liver transplantation, sarcopenia, rocuronium, neuromuscular blockade, pharmacodynamics, train-of-four

## Abstract

**Background**: Sarcopenia is highly prevalent in end-stage liver disease and is associated with adverse perioperative outcomes. However, its association with rocuronium pharmacodynamics during liver transplantation (LT) remains insufficiently defined. **Aim**: This study aimed to evaluate the association between sarcopenia, neuromuscular blockade kinetics, and clinical outcomes in LT recipients. **Methods**: In this prospective observational cohort study, 139 adult LT recipients were classified as sarcopenic (*n* = 70) or non-sarcopenic (*n* = 69) based on EWGSOP2 criteria, including SARC-F, handgrip strength, and psoas muscle index (PMI). Rocuronium (1 mg/kg, ideal body weight) was administered at induction, and quantitative neuromuscular monitoring was performed using train-of-four (TOF). The primary outcome was time to complete neuromuscular blockade (T0). Secondary outcomes included intraoperative neuromuscular recovery parameters, perioperative clinical variables, and postoperative outcomes. Multivariable GLM analyses were performed to evaluate factors associated with sarcopenia and T0, while logistic regression models were used to assess factors associated with mortality. **Results**: Sarcopenic patients exhibited significantly reduced PMI (*p* < 0.001) and lower handgrip strength (*p* = 0.001). In the baseline binomial-logit GLM, age was independently associated with sarcopenia (OR = 1.034, *p* = 0.025). The onset of neuromuscular blockade was significantly prolonged in the sarcopenic group (T0: 100 vs. 80 s; *p* < 0.001). In the adjusted Gamma regression model, sarcopenia remained significantly associated with longer T0 after adjustment for age, sex, MELD score, BMI, and hemoglobin level (adjusted ratio = 1.232, 95% CI: 1.105–1.372, *p* < 0.001). Postoperatively, they demonstrated prolonged extubation time (10 vs. 7 h; *p* < 0.001), extended ICU stay (9 vs. 6 days; *p* < 0.001), and higher mortality (27.1% vs. 8.7%; *p* = 0.009). In multivariable logistic regression, sarcopenia was independently associated with mortality (OR = 3.26; *p* = 0.023), while each additional ICU day was associated with an approximate 9% increase in mortality risk in the secondary model. **Conclusions**: Sarcopenia was associated with altered rocuronium pharmacodynamics in LT recipients, primarily characterized by delayed onset of complete neuromuscular blockade, and this association persisted after adjustment for age and other baseline clinical variables. Sarcopenic recipients also showed prolonged extubation time, longer ICU stay, and higher mortality. These findings support the integration of sarcopenia into perioperative risk stratification and individualized neuromuscular management strategies in this high-risk population.

## 1. Introduction

Since the first successful liver transplantation (LT) performed by Thomas Starzl and colleagues in 1967, LT has evolved into a well-established, life-saving therapy for patients with end-stage liver disease (ESLD), including decompensated cirrhosis or chronic liver failure, as well as acute liver failure and selected metabolic disorders [1,2]. Continuous advances in surgical techniques, perioperative management, and immunosuppressive strategies have markedly improved post-LT survival, resulting in substantial gains in graft function and long-term patient outcomes [3,4]. As survival improves, perioperative determinants of functional recovery and physiological resilience have become increasingly relevant in LT recipients. Among these clinically relevant perioperative risk modifiers, sarcopenia—closely linked to frailty and diminished muscle reserve—has emerged as a clinically relevant factor associated with perioperative outcomes in LT recipients.

Sarcopenia has emerged as a highly prevalent and clinically significant condition in patients with ESLD, affecting approximately 30–70% of candidates awaiting LT and being associated with a 1.84-fold higher post-transplant mortality risk [5,6,7,8]. Tandon et al. [9] demonstrated that severe muscle depletion is an independent prognostic factor in patients on the LT waiting list, while the systematic review and meta-analysis by van Vugt et al. [10] confirmed that low skeletal muscle mass, as assessed by computed tomography (CT), is directly associated with adverse LT outcomes. Beyond its established association with adverse outcomes, accumulating evidence suggests that pretransplant sarcopenia may also be associated with altered perioperative physiological responses [11]. Accordingly, severe muscle depletion has been recognized not only as a prognostic marker but also as a potential marker of perioperative pharmacological and physiological variability in LT recipients.

These clinical effects are underpinned by multifactorial mechanisms involving impaired protein metabolism, neuromuscular junction degeneration, chronic systemic inflammation, and endocrine dysfunction [8,12,13,14]. In ESLD, additional disease-specific processes such as hyperammonemia-induced autophagy, inhibition of the mechanistic target of rapamycin complex 1 (mTORC1) pathway, and upregulation of myostatin further accelerate muscle wasting [15,16,17]. Of particular relevance, in sarcopenia, denervation resulting from motor neuron loss leads to the upregulation of fetal-type (γ-subunit–containing) acetylcholine receptors at the postsynaptic membrane. Compared with adult-type (ε-subunit-containing) receptors, these exhibit lower affinity for non-depolarizing agents, suggesting a plausible mechanistic basis for altered responsiveness to neuromuscular blocking agents [18,19].

Rocuronium is a widely used intermediate-acting aminosteroid non-depolarizing neuromuscular blocking agent [20]. Approximately 70% of its elimination occurs via hepatic pathways; therefore, in liver failure, its volume of distribution increases and clearance is reduced [20,21,22]. However, current dosing protocols are primarily based on ideal body weight (IBW) and do not account for variations in muscle mass or quality [23,24]. In sarcopenic patients, reduced muscle mass, altered extracellular fluid distribution, and neuromuscular junction remodeling collectively contribute to altered drug distribution and neuromuscular transmission [14,25]. These alterations suggest that conventional IBW-based dosing may not adequately reflect pharmacodynamic variability in this population. Accordingly, the pharmacodynamic profile of rocuronium may differ in this population, potentially affecting both onset and recovery characteristics [25].

The literature directly investigating the relationship between sarcopenia and neuromuscular blocking agents remains extremely limited. Murphy et al. [26] demonstrated an increased incidence of postoperative residual neuromuscular blockade in elderly patients with reduced muscle mass; however, no prospective study has specifically evaluated the association between sarcopenia and rocuronium pharmacodynamics using objective train-of-four (TOF) monitoring in liver transplant recipients. This represents a critical gap in perioperative pharmacology. Notably, reduced muscle mass might intuitively be expected to accelerate drug onset due to a smaller distribution volume; however, emerging physiological considerations suggest that sarcopenia may be associated with delayed onset through receptor-level and perfusion-related mechanisms. Based on this conceptual and clinical gap, the present study aims to evaluate the association between sarcopenia and rocuronium onset time, intraoperative neuromuscular blockade kinetics, and postoperative clinical outcomes in LT recipients.

## 2. Materials and Methods

### 2.1. Study Protocol, Ethical Considerations, and Funding

This prospective observational cohort study was conducted at the Inonu University Liver Transplant Institute (Malatya, Türkiye). All procedures were performed in accordance with the ethical principles of the Declaration of Helsinki (1964) and its subsequent amendments, and complied with all applicable institutional and national regulations governing research involving human participants. Written informed consent was obtained from all participants prior to the LT procedure. Ethical approval was obtained from the Inonu University Clinical Research Ethics Committee on 11 March 2025 (Approval number: 7237). Following ethical approval and before enrollment of LT recipients, the study record was submitted to ClinicalTrials.gov on 20 March 2025 (NCT06909942), subsequently met quality-control criteria, and was publicly posted on 4 April 2025. Enrollment of LT recipients and study-specific data collection were initiated only after completion of the relevant ethical and registration procedures. Publication-related financial support was obtained from the Inonu University Scientific Research Projects Coordination Unit (Grant ID: TSA-2026-4807) solely to cover the article processing charge or open access publication fee after manuscript acceptance. This support was not used for patient enrollment, data collection, data analysis, or any stage of study conduct. The study was designed, conducted, analyzed, and reported in accordance with the STROBE (Strengthening the Reporting of Observational Studies in Epidemiology) guidelines to enhance transparency, reproducibility, and methodological rigor.

### 2.2. A Priori Power Analysis

A priori sample size calculation was performed. At a significance level of 5% and a statistical power of 80%, with an effect size of 0.701 and a two-tailed hypothesis, a minimum sample size of 70 patients (35 per group) was required to detect a statistically significant difference in T0 between the non-sarcopenic and sarcopenic groups using the Mann–Whitney U test. Power calculations were performed using WSSPAS (Web-Based Sample Size and Power Analysis Software, version 2.0).

### 2.3. Study Population and Patient Selection

Patients aged 18–65 years with ASA III–IV classification who were scheduled for elective liver transplantation at the Inonu University Liver Transplant Institute and who had provided informed consent to undergo LT were included in the study. Exclusion criteria comprised inability to provide informed consent, age < 18 or >65 years, advanced renal failure requiring renal replacement therapy, massive ascites, severe cardiac failure or pulmonary hypertension necessitating deviation from standard anesthesia protocols, psychiatric disorders or substance abuse, acute liver failure, body mass index (BMI) > 40 kg/m^2^, inconclusive sarcopenia classification, intraoperative massive transfusion, and known hypersensitivity to rocuronium or sugammadex. Inconclusive sarcopenia classification was defined as inconsistent findings across the three sarcopenia assessment components described in Section 2.4, namely the SARC-F (Strength, Assistance in walking, Rise from a chair, Climb stairs, and Falls) questionnaire, handgrip strength measurement, and CT-based psoas muscle index (PMI), which precluded assignment to either the sarcopenia or non-sarcopenia group.

### 2.4. Sarcopenia Assessment

Sarcopenia was diagnosed using a three-step algorithm based on European Working Group on Sarcopenia in Older People 2 (EWGSOP2) criteria [12,14]: (i) the SARC-F questionnaire, a five-domain screening tool scored from 0 to 10, with a score ≥ 4 considered indicative of sarcopenia; (ii) handgrip strength, measured using a handheld dynamometer (Camry Digital Hand Dynamometer, Camry Scale, South El Monte, CA, USA), with cut-off values defined as <27.9 kg for men and <16.7 kg for women; and (iii) PMI, calculated from abdominal CT scans obtained within 4 weeks prior to LT. For PMI calculation, the cross-sectional areas of the right and left psoas muscles at the L3 vertebral level were measured and normalized to height squared (m^2^), using sex-specific cut-off values derived from the Turkish population [27]. All CT measurements were performed by the same radiologist using standardized imaging software (SECTRA IDS 7 (Sectra AB, Linköping, Sweden)) and predefined window settings to ensure consistency and reproducibility. LT recipients were assigned to the sarcopenia group when SARC-F score was ≥4, handgrip strength was reduced, and PMI was below the sex-specific cut-off. Recipients were assigned to the non-sarcopenia group when SARC-F score was <4, handgrip strength was preserved, and PMI was above the sex-specific cut-off. Discordant results among SARC-F, handgrip strength, and PMI were considered inconclusive sarcopenia classification.

### 2.5. Anesthesia Protocol

IBW was calculated using the Devine formula. All patients underwent preoperative assessment including handgrip strength measurement and SARC-F scoring. Standard intraoperative monitoring included five-lead ECG, pulse oximetry, invasive arterial blood pressure monitoring, bispectral index (BIS), and quantitative neuromuscular monitoring using TOF-Watch SX (Organon, Jersey City, NJ, USA). Anesthesia induction was performed with thiopental (5–7 mg/kg IV, based on IBW), fentanyl (1–2 µg/kg IV, based on IBW), and rocuronium (1 mg/kg IV, based on IBW). All induction agents were administered according to IBW as part of the standardized institutional anesthesia protocol for LT recipients, aiming to reduce variability from actual body weight, which may be substantially influenced by ascites, edema, fluid retention, and altered body composition [28,29,30,31]. The time to complete neuromuscular blockade (T0; TOF = 0) was recorded in seconds. A modified rapid sequence induction (mRSI) protocol was applied due to aspiration risk. After adequate preoxygenation, brief low-pressure mask ventilation (<20 cmH_2_O) was allowed. Intubation conditions were evaluated using the Modified Helbo-Hansen Raulo scoring system (5–20 points) [32].

During TOF monitoring, recovery of the first twitch response to 30% of baseline, observation of at least three twitches in the TOF sequence, or triggering of spontaneous respiration on the mechanical ventilator at a threshold of ≥3 cmH_2_O was accepted as evidence indicating resolution of neuromuscular blockade. Accordingly, the first time point at which any of these findings was observed was defined as T1, the second time point as T2, and the third time point as T3. At each of these time points, an additional dose of rocuronium (0.15 mg/kg) was administered. The surgical phase corresponding to each dosing event (dissection, anhepatic, or neohepatic phase) was systematically documented. Perioperative temperature management was maintained using forced-air warming blankets, and all intravenous fluids were delivered through a fluid-warming system (ICU Medical Hotline). Anesthetic depth was standardized using BIS monitoring with a target range of 40–60, and maintenance anesthesia was conducted using a standardized volatile anesthetic protocol.

### 2.6. Immunosuppressive Protocol

Immunosuppression followed a standardized institutional protocol: induction with 500 mg intravenous methylprednisolone administered intraoperatively after hepatic artery anastomosis, followed by tapering over the first 10 postoperative days and short-term maintenance with low-dose corticosteroids up to 3 months (discontinued thereafter in the absence of specific indications). Mycophenolate mofetil (500–1000 mg twice daily) was initiated early postoperatively and typically discontinued at 6 months. Tacrolimus was started on postoperative day 3 (target trough 8–10 ng/mL), with delayed initiation and basiliximab bridging in cases of perioperative renal dysfunction; tacrolimus-sparing strategies were used if renal recovery was inadequate. None of the included patients had hepatorenal syndrome or perioperative renal impairment.

### 2.7. Postoperative Management and Routine Follow-Up

After LT procedures, all recipients were transferred to the intensive care unit (ICU) under mechanical ventilation. Quantitative neuromuscular monitoring was continued in the ICU, and extubation was performed after sugammadex reversal (2 mg/kg) when standard clinical and respiratory criteria were fulfilled [33]. Time to extubation, ICU length of stay, and total hospital length of stay were recorded. Post-discharge follow-up was conducted according to the institutional LT surveillance protocol, which includes regular outpatient visits during the first postoperative year. Follow-up data were obtained from hospital records, outpatient clinic documentation, and, when required, the most recent verified patient or family contact. For non-survivors, follow-up time was defined as the interval from LT to death; for survivors, it was defined as the interval from LT to the last outpatient evaluation or verified contact. In the present study, follow-up data were reviewed at 1, 3, and 6 months after LT, and 12-month data were included only for LT recipients who had completed this interval by the database lock date.

### 2.8. Study Endpoints

The primary endpoint was rocuronium onset time, defined as the interval from completion of rocuronium administration to complete neuromuscular blockade (T0; TOF = 0), recorded in seconds. Secondary endpoints were predefined to characterize intraoperative neuromuscular blockade kinetics, perioperative clinical course, and postoperative follow-up findings. These included neuromuscular recovery parameters (T1, T2, and T3), phase-specific recovery patterns, intubation score, duration of surgery, intraoperative erythrocyte suspension transfusion requirement, time to extubation, ICU length of stay, total hospital length of stay, and mortality during postoperative follow-up.

### 2.9. Statistical Analysis

Statistical analyses were performed using IBM SPSS Statistics for Windows, Version 27.0 (IBM Corp., Armonk, NY, USA). Graphical representations were generated using GraphPad Prism version 10.6.1 (GraphPad Software, San Diego, CA, USA). Continuous variables were summarized as median (25th–75th percentiles), and categorical variables as frequency and percentage. The Shapiro–Wilk test was used to assess normality. Because most continuous variables were not normally distributed, between-group comparisons were performed using the Mann–Whitney U test. Categorical variables were compared using the Pearson chi-square test or Fisher’s exact test, as appropriate.

To improve the clinical interpretability of between-group comparisons, effect estimates were reported together with 95% confidence intervals (CIs). For continuous variables, unadjusted effect estimates were calculated as median differences with 95% CIs using median quantile regression at q = 0.50. For comparisons according to sarcopenia status, median differences were calculated as sarcopenic minus non-sarcopenic. For comparisons according to survival status, median differences were calculated as non-survivors minus survivors. For categorical variables, crude odds ratios (ORs) with 95% CIs were reported. These effect estimates were used to complement *p*-values and to describe the magnitude and direction of observed differences.

To identify baseline clinical factors associated with sarcopenia, a generalized linear model (GLM) with binomial distribution and logit link function was constructed, with sarcopenia status as the dependent variable. Age, BMI, MELD score, and hemoglobin level were included as independent variables based on clinical relevance and prior literature. To further examine whether the prolongation in rocuronium onset time was independently associated with sarcopenia after accounting for potential baseline confounders, an additional multivariable GLM with Gamma distribution and log link function was constructed using T0 as the dependent variable. Sarcopenia status, age, sex, MELD score, BMI, and hemoglobin level were included as independent variables. Results from this model were reported as adjusted ratios derived from Exp(B), with 95% CIs. Muscle-related variables used to define sarcopenia, including PMA, PMI, and handgrip strength, were not included in this model to avoid conceptual overlap and collinearity.

For mortality analysis, variables with a *p*-value < 0.05 in univariate analyses were included in a separate multivariable logistic regression model using the enter method. Results were reported as ORs with 95% CIs. Two models were constructed: a primary model including baseline clinical variables and a secondary model additionally including ICU length of stay. ICU length of stay was analyzed separately because it reflects the postoperative clinical course and may act as a mediator rather than an independent baseline predictor.

Given the number of comparisons performed across baseline characteristics, intraoperative neuromuscular blockade parameters, surgical-phase recovery patterns, and postoperative outcomes, no formal multiplicity adjustment was applied. Therefore, *p*-values from secondary comparisons were interpreted cautiously because of the potential risk of type I error. A two-sided *p*-value < 0.05 was considered statistically significant.

## 3. Results

### 3.1. Patient Characteristics and Prevalence of Sarcopenia

During the study period, 161 patients were assessed for eligibility. Of these, 10 were excluded due to intraoperative massive hemorrhage and 12 due to the need for postoperative reoperation, resulting in a final cohort of 139 patients. The majority of patients were male (71.2%, *n* = 99), and all were classified as ASA III–IV. The prevalence of sarcopenia was 50.4% (*n* = 70). The most common etiologies of ESLD were cryptogenic cirrhosis (25.2%), hepatitis B virus infection (16.5%), and autoimmune hepatitis (7.2%).

Table 1 demonstrates the detailed demographic and preoperative characteristics of the study population. Compared with the non-sarcopenic group, patients with sarcopenia were significantly older (54.0 vs. 47.0; *p* = 0.025) and had lower body weight (69.0 vs. 78.0; *p* = 0.031) and BMI (23.8 vs. 26.7; *p* = 0.041). The median MELD score was significantly higher in the sarcopenic group (18.0 vs. 16.0; *p* = 0.045), whereas Child–Pugh scores were comparable (*p* = 0.157). Hemoglobin levels were significantly lower in the sarcopenic group (9.9 vs. 11.2; *p* = 0.021).

To identify baseline clinical factors associated with sarcopenia, a multivariable GLM analysis with binomial distribution and logit link was performed. Sarcopenia status was entered as the dependent variable, while age, BMI, MELD score, and hemoglobin level were included as independent variables. In this adjusted model, age was the only variable independently associated with sarcopenia (B = 0.033, OR = 1.034, 95% CI: 1.004–1.064, *p* = 0.025). This indicates that each one-year increase in age was associated with an approximately 3.4% increase in the odds of sarcopenia, after controlling for BMI, MELD score, and hemoglobin level. By contrast, BMI showed a negative but statistically non-significant association with sarcopenia (B = −0.059, OR = 0.942, *p* = 0.066). Similarly, MELD score was not significantly associated with sarcopenia in the adjusted model (B = 0.048, OR = 1.049, *p* = 0.152), nor was hemoglobin level (B = −0.104, OR = 0.901, *p* = 0.175). These findings suggest that, among the baseline variables included in the model, age had the most consistent independent association with sarcopenic status in this cohort.

As expected, sarcopenia-defining parameters demonstrated marked and clinically meaningful differences between groups. The PMA was significantly lower in the sarcopenic group (9.0 vs. 18.5; *p* < 0.001). Similarly, the PMI was significantly lower (3.0 vs. 6.0; *p* < 0.001), indicating substantial depletion of skeletal muscle mass. Handgrip strength was also profoundly reduced in sarcopenic patients (13.0 vs. 40.0; *p* = 0.001), reflecting severe impairment in muscle function. These findings confirm that both structural (PMA, PMI) and functional (handgrip strength) components of sarcopenia were significantly compromised and constituted the primary discriminative features between groups.

### 3.2. Intraoperative Neuromuscular Blockade Parameters

**Figure 1:**
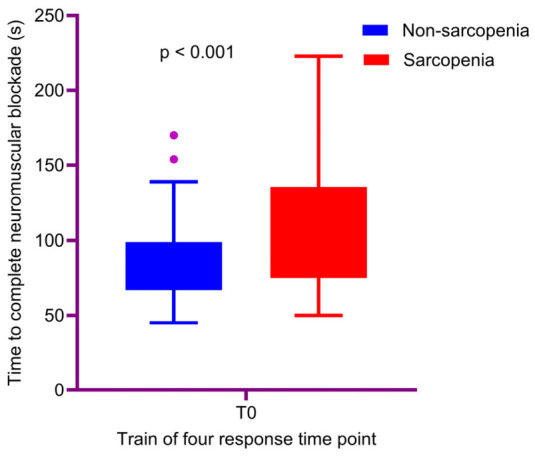
Comparison of time to complete neuromuscular blockade (T0) between sarcopenic and non-sarcopenic LT recipients. Data are presented as medians with interquartile ranges. The purple dots represent outliers outside the interquartile range.

**Figure 2:**
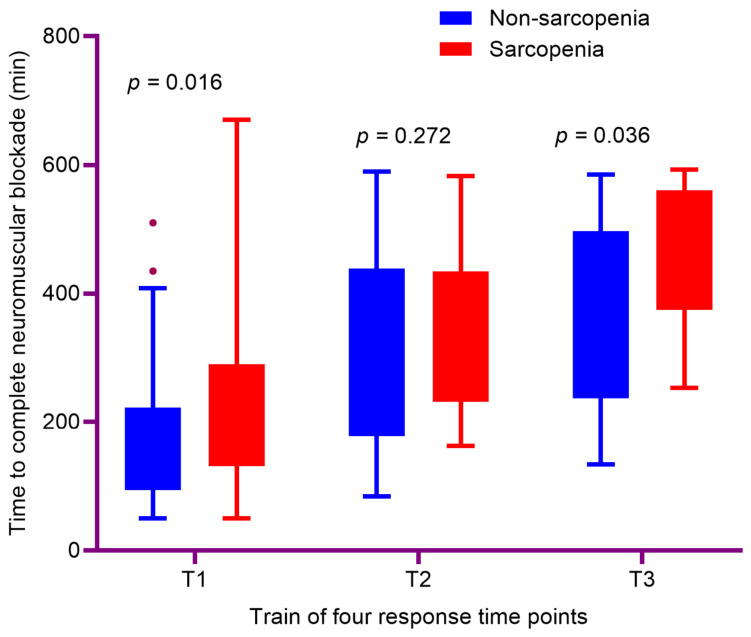
Comparison of intraoperative neuromuscular recovery/redosing time points (T1–T3) between sarcopenic and non-sarcopenic LT recipients. Data are presented as medians with interquartile ranges. The purple dots represent outliers outside the interquartile range.

Table 2 demonstrates the intraoperative TOF parameters and associated surgical characteristics of the study population. The time to complete neuromuscular blockade (T0) was significantly prolonged in the sarcopenic group (100 vs. 80 s; *p* < 0.001) (Figure 1). Likewise, the time to first recovery of TOF to ≥30% (T1) was significantly longer in the sarcopenic group (192 vs. 165 min; *p* = 0.016). Although T2 was numerically longer in the sarcopenic group (330 vs. 263 min; *p* = 0.272), this difference was not statistically significant. In contrast, T3 was significantly prolonged in the sarcopenic group (420 vs. 345 min; *p* = 0.036) (Figure 2).

**Figure 3:**
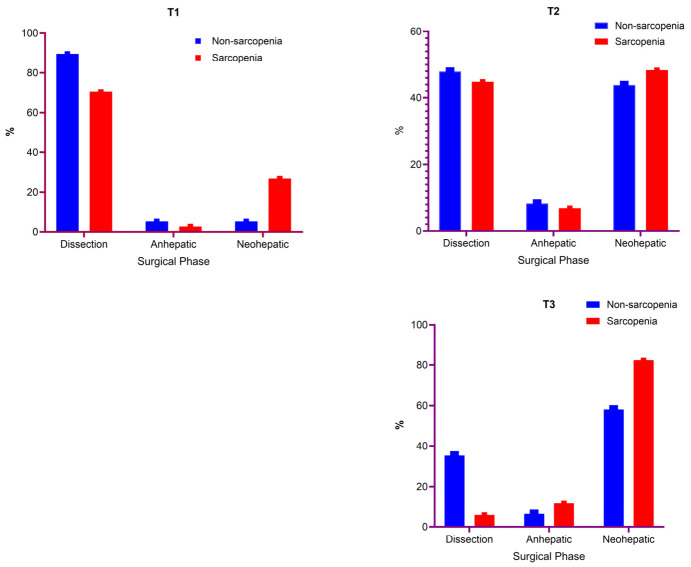
Phase-specific distribution (%) of intraoperative neuromuscular recovery/redosing time points (T1–T3) across surgical phases in sarcopenic versus non-sarcopenic LT recipients.

When the timing of TOF recovery was examined according to surgical phase, T1 during the dissection phase was more frequent in the non-sarcopenic group (89.5 vs. 69.4%; *p* = 0.031), whereas T1 during the neohepatic phase was more frequent in the sarcopenic group (27.8 vs. 5.3%; *p* = 0.004). No significant between-group differences were found for T2 during either the dissection phase (*p* = 1.000) or the neohepatic phase (*p* = 1.000). For T3, recovery during the dissection phase was significantly less frequent in the sarcopenic group (5.9% vs. 35.5%; *p* = 0.035), whereas T3 during the neohepatic phase was numerically higher in the sarcopenic group but did not reach statistical significance (*p* = 0.165) (Figure 3).

The intubation score was significantly lower in the sarcopenic group (median 5 vs. 5; mean ± SD 5.6 ± 0.89 vs. 5.3 ± 0.92; *p* = 0.037), indicating better intubation conditions. Intraoperative erythrocyte suspension transfusion requirements were higher in the sarcopenic group (4 vs. 2 units; *p* = 0.054), although this difference did not reach statistical significance. In addition, the duration of surgery was longer in the sarcopenic group (11 vs. 10 h; *p* = 0.040).

To further examine whether the observed prolongation in rocuronium onset time could be explained by age or other baseline clinical differences between groups, a multivariable Gamma regression model with log link was constructed using T0 as the dependent variable. Sarcopenia status, age, sex, MELD score, BMI, and hemoglobin level were included as independent variables. The model included 139 recipients with complete covariate data and was statistically significant overall (likelihood ratio χ^2^ = 30.454, df = 6, *p* < 0.001). In this adjusted model, sarcopenia status remained significantly associated with T0 (Wald χ^2^ = 14.273, *p* < 0.001), whereas age (*p* = 0.386), sex (*p* = 0.289), MELD score (*p* = 0.243), BMI (*p* = 0.352), and hemoglobin level (*p* = 0.097) were not independently associated with T0. Expressed in the clinically intuitive direction, sarcopenic recipients had a 23.2% longer T0 than non-sarcopenic recipients after adjustment for these covariates (adjusted ratio = 1.232, 95% CI: 1.105–1.372). These findings suggest that the delayed onset of complete neuromuscular blockade in sarcopenic recipients was not explained solely by chronological age or by the other baseline clinical differences included in the model.

### 3.3. Postoperative Clinical Outcomes

Table 3 summarizes the postoperative clinical outcomes of the study population. ICU length of stay and time to extubation were significantly higher in the sarcopenic group compared with the non-sarcopenic group (9.0 vs. 6.0; *p* < 0.001; and 10 vs. 7; *p* < 0.001, respectively). In contrast, total hospital length of stay did not differ significantly between the groups (29 vs. 30; *p* = 0.831). Postoperative mortality was also significantly higher in the sarcopenic group (27.1% vs. 8.7%; *p* = 0.009).

### 3.4. Comparison of Survivors and Non-Survivors

Table 4 demonstrates the comparison of survivors and non-survivors according to baseline, intraoperative, and postoperative variables. Compared with survivors, non-survivors had significantly lower PMA (9.40 vs. 14.1; *p* = 0.004), PMI (3.2 vs. 5.0; *p* = 0.003), handgrip strength (15 vs. 32; *p* = 0.009), and hemoglobin levels (9.0 vs. 10.9; *p* = 0.003). In contrast, total bilirubin and direct bilirubin levels were significantly higher in non-survivors (4.3 vs. 3.0; *p* = 0.014; and 2.1 vs. 1.1; *p* = 0.031, respectively). Among intraoperative parameters, T3 was significantly prolonged in non-survivors (495.0 vs. 375; *p* = 0.014), and ES transfusion was significantly higher (4 vs. 3 units; *p* = 0.019). In the postoperative period, ICU length of stay was significantly longer in non-survivors (14 vs. 6.5; *p* < 0.001), whereas hospital length of stay was significantly shorter (19 vs. 30; *p* = 0.002).

### 3.5. Multivariable Analysis of Factors Associated with Mortality

Table 5 demonstrates the results of the multivariable logistic regression analysis of factors associated with mortality. In the primary model including sarcopenia, hemoglobin, and total bilirubin, the overall model was statistically significant (Omnibus test *p* = 0.001) and showed acceptable goodness-of-fit (Hosmer–Lemeshow *p* = 0.238). The model explained a modest proportion of the variance in mortality (Nagelkerke R^2^ = 0.185). Sarcopenia was independently associated with increased mortality (OR = 3.26; *p* = 0.023). Hemoglobin was inversely associated with mortality (OR = 0.80; *p* = 0.046), indicating that higher hemoglobin levels were associated with lower mortality odds. Total bilirubin was not independently associated with mortality (OR = 1.06; *p* = 0.213). In the secondary model, ICU length of stay was strongly associated with mortality (OR = 1.09; *p* < 0.001), corresponding to an approximately 9% increase in mortality risk per additional ICU day. This variable was analyzed separately as it reflects postoperative clinical course and may act as a mediator rather than an independent baseline risk factor. To specifically address baseline liver disease severity as reflected by MELD score, an additional sensitivity analysis was performed using a GLM with binomial distribution and logit link. In this model, sarcopenia remained significantly associated with mortality after adjustment for MELD score and hemoglobin level (OR = 3.34, 95% CI: 1.21–9.26, *p* = 0.020), whereas MELD score was not independently associated with mortality (OR = 1.02, 95% CI: 0.94–1.10, *p* = 0.670). Hemoglobin retained an inverse association with mortality (OR = 0.78, 95% CI: 0.63–0.97, *p* = 0.024), suggesting that the observed mortality difference was not explained solely by baseline liver disease severity.

## 4. Discussion

This prospective observational study found that, in sarcopenic LT recipients, the onset time of rocuronium-induced neuromuscular blockade (T0) was significantly prolonged and that intraoperative blockade kinetics were meaningfully altered. Furthermore, sarcopenic patients exhibited clinically relevant postoperative differences, including prolonged time to extubation, extended ICU length of stay, and increased postoperative mortality. These findings highlight a clinically important association between sarcopenia, altered neuromuscular pharmacodynamics, and adverse perioperative outcomes in LT recipients [6,34].

The observed sarcopenia prevalence of 50.4% in the present cohort is consistent with the reported 30–70% range in patients with ESLD [5,6,34,35]. This high prevalence primarily reflects the disease-specific pathophysiology of liver failure rather than general population risk factors. In ESLD, hyperammonemia-driven autophagy and inhibition of mTORC1 signaling accelerate muscle protein breakdown, while portal hypertension–related malabsorption, chronic inflammation, and malnutrition—often exacerbated by ascites—impair muscle protein synthesis [15,34]. In addition, reduced hepatic metabolic reserve and systemic inflammatory burden contribute to a progressive decline in muscle quantity and quality, placing these patients along a frailty–sarcopenia continuum. Collectively, these mechanisms may help explain why sarcopenia is highly prevalent and clinically impactful in LT candidates, beyond traditional lifestyle-associated risk factors [36,37].

The three-step EWGSOP2-based algorithm used for the diagnosis of sarcopenia yielded notable findings regarding its applicability in this patient population [38,39]. Meta-analyses have consistently demonstrated that a SARC-F score ≥ 4 has low-to-moderate sensitivity but moderate-to-high specificity for sarcopenia screening, indicating that it is more useful for identifying high-risk individuals than for excluding the condition [40,41,42]. Accordingly, SARC-F should not be used as a stand-alone screening tool but rather interpreted alongside objective measures such as muscle strength and imaging-based indices. Regarding muscle mass assessment, PMI represents a practical and relatively less confounded metric in liver disease, as the psoas muscle is less affected by ascites and peripheral edema compared with broader muscle measurements. In the present study, the markedly lower PMI values in the sarcopenic group indicate that this measurement effectively captured between-group differences in skeletal muscle depletion. Previous studies have reported an association between low PMI and increased mortality risk in patients with ESLD [43]. In line with this evidence, the higher mortality observed in the sarcopenic group in the present study further supports the clinical relevance of CT-based muscle assessment in LT recipients. Although some reports suggest that sarcopenia is more common in men than in women [44], the present study found no significant sex-based difference in sarcopenia prevalence. The influence of sex on sarcopenia in cirrhotic patients remains controversial, and larger well-designed prospective studies are needed in this area.

Aging and chronic disease burden are well-established drivers of both sarcopenia and physical frailty, reflecting a progressive decline in physiological reserve and increased vulnerability to perioperative stressors [45,46]. In the present study, the older age distribution observed in sarcopenic LT recipients was consistent with this established relationship. Age remained independently associated with sarcopenic status after adjustment for BMI, MELD score, and hemoglobin level, supporting the close biological link between aging and sarcopenia in this population. This finding is biologically plausible, as aging is characterized by reduced anabolic reserve, impaired myofibrillar protein synthesis, hormonal decline, chronic low-grade inflammation, and progressive deterioration in neuromuscular integrity [47].

Importantly, age may also influence rocuronium-related neuromuscular blockade kinetics and postoperative recovery [20,48,49]. Therefore, the present study further examined whether the delayed onset of complete neuromuscular blockade observed in sarcopenic recipients could be explained by chronological age or other baseline clinical differences. In the adjusted model, sarcopenia status remained associated with T0 after accounting for age, sex, MELD score, BMI, and hemoglobin level, whereas age itself was not independently associated with T0. These findings suggest that the observed T0 prolongation was not explained solely by chronological age. Nevertheless, biological aging and sarcopenia are closely interrelated and cannot be completely disentangled in an observational cohort. Therefore, the observed alterations in rocuronium-related neuromuscular blockade kinetics and postoperative recovery should not be interpreted as exclusively sarcopenia-specific, but rather as findings that may reflect the combined influence of sarcopenia, aging-related physiological decline, reduced neuromuscular reserve, impaired tissue perfusion, respiratory muscle vulnerability, and diminished postoperative resilience.

Having established the clinical relevance and assessment framework of sarcopenia in this cohort, the most distinctive finding of the present study was its association with altered rocuronium onset and recovery kinetics. Although previous studies have reported prolonged rocuronium onset and duration in older populations, to the best of current knowledge, no study has directly evaluated the relationship between sarcopenia and rocuronium pharmacodynamics [50]. The initial hypothesis of the present study was that reduced muscle mass in sarcopenic patients would lead to a smaller volume of distribution and consequently a faster onset of blockade. However, the findings of the present study did not support this hypothesis; on the contrary, T0 was significantly prolonged in the sarcopenic group, with delays reaching up to 223 s in some cases. This unexpected finding should be interpreted within the combined pathophysiological context of sarcopenia and ESLD rather than as an isolated effect of reduced muscle mass.

Several biologically plausible mechanisms may help explain the delayed onset and altered recovery pattern observed in sarcopenic LT recipients. First, structural and functional remodeling at the neuromuscular junction may contribute to altered responsiveness to non-depolarizing neuromuscular blocking agents. Sarcopenia-related denervation may disrupt postsynaptic junctional architecture and promote upregulation of extrasynaptic fetal-type acetylcholine receptors; compared with adult-type receptors, these receptors differ structurally and may show lower affinity for non-depolarizing agents [51,52]. Second, expansion of the volume of distribution due to increased extracellular fluid in liver failure may delay effective drug delivery. Classic pharmacokinetic data by Khalil et al. [21] demonstrated a marked increase in steady-state volume of distribution in cirrhotic patients (0.21 L/kg to 0.38 L/kg), supporting this mechanism. Third, reduced capillary density and impaired peripheral perfusion in sarcopenic muscle may delay delivery of neuromuscular blocking agents to the neuromuscular junction, as their onset and effect are influenced by muscle perfusion and distribution characteristics [25,53]. Finally, the bidirectional interaction between sarcopenia and end-stage liver disease—where liver dysfunction promotes muscle wasting and sarcopenia reduces metabolic reserve—may amplify these pharmacodynamic alterations.

In addition to these biological mechanisms, the dosing strategy should also be considered when interpreting the pharmacodynamic findings. Rocuronium dosing was standardized according to IBW in the present study to reduce dose variability related to actual body weight in LT recipients, in whom ascites, edema, fluid retention, and altered body composition may distort weight-based dosing. However, the choice of dosing scalar remains an important issue in anesthetic pharmacology. Actual body weight may overestimate the pharmacologically relevant distribution compartment in patients with fluid overload, whereas adjusted or corrected body weight has mainly been evaluated in obese populations and has not been established as a universally accepted dosing approach for sarcopenic LT recipients. Therefore, although IBW-based dosing allowed protocol standardization between groups, it may not fully account for interindividual differences in skeletal muscle mass and body composition. This limitation should be considered when interpreting the observed differences in rocuronium onset and neuromuscular blockade kinetics.

The phase-specific recovery findings further support the presence of altered intraoperative blockade kinetics in sarcopenic recipients. Spontaneous TOF recovery was shifted toward later surgical phases compared with non-sarcopenic patients, suggesting a more sustained blockade profile. This pattern may reflect the combined influence of sarcopenia-related changes in muscle mass, neuromuscular junction structure, tissue perfusion, and extracellular fluid distribution, together with phase-dependent hepatic function during LT. Because rocuronium is predominantly eliminated via the hepatobiliary system, impaired liver function during the dissection phase and markedly reduced hepatic clearance during the anhepatic phase may prolong blockade, whereas gradual restoration of graft function during the neohepatic phase may facilitate recovery [54]. Thus, the clustering of T3 recordings in the neohepatic phase in sarcopenic recipients may reflect this physiological transition rather than a sarcopenia-specific effect alone. These findings emphasize the need for careful rocuronium titration throughout all surgical phases and the use of quantitative neuromuscular monitoring in sarcopenic LT recipients.

The prolonged T0 observed in sarcopenic recipients also has practical implications for airway management. Given the high aspiration risk in this population, rapid sequence induction (RSI) remains clinically relevant [55]; however, in the present study, laryngoscopy and tracheal intubation were performed only after TOF reached 0 in all patients. Therefore, the longer T0 in the sarcopenic group should be interpreted as delayed TOF-defined readiness for intubation, rather than as poorer intubation conditions at the actual time of laryngoscopy. This distinction may explain why prolonged T0 could coexist with statistically lower intubation scores. The intubation score represents a clinical composite assessment performed after complete blockade had been achieved, not a direct surrogate of rocuronium onset speed. Moreover, because the median intubation score was 5 in both groups, this finding should be interpreted as a small difference in clinical scoring rather than as evidence that sarcopenia improves intubation conditions. One possible explanation is that reduced skeletal muscle mass and soft-tissue volume in sarcopenic recipients may have contributed to more favorable mechanical conditions during laryngoscopy, particularly through components related to jaw relaxation and muscular resistance; however, this interpretation should be regarded as hypothesis-generating rather than definitive. To our knowledge, no specific literature has directly examined the relationship between sarcopenia, intubation conditions, and TOF-defined neuromuscular blockade depth. Therefore, further studies are needed to evaluate the anatomical and pharmacodynamic variables contributing to intubation scoring in sarcopenic patients.

Beyond induction and intubation timing, delayed neuromuscular recovery also has implications for postoperative extubation and residual blockade risk. Residual neuromuscular blockade (RNMB), defined as a TOF ratio < 0.9, remains a significant patient safety concern in anesthetic practice. The 2023 ASA Neuromuscular Blockade Guidelines strongly recommend achieving a TOF ratio ≥ 0.9 prior to extubation and consider reversal without quantitative monitoring unacceptable [56]. The ESA 2023 guidelines similarly support this recommendation [57]. Current literature demonstrates a strong association between RNMB and adverse respiratory events, including aspiration pneumonia, atelectasis, hypoventilation, and critical respiratory complications [58]. In the present cohort, the prolonged extubation time observed in sarcopenic patients may reflect the combined effect of delayed neuromuscular recovery and reduced respiratory muscle reserve. Accordingly, routine quantitative monitoring and standardized sugammadex reversal were central components of postoperative management in this high-risk population. However, the present study did not specifically evaluate sugammadex reversal kinetics, such as time from sugammadex administration to TOF ratio ≥ 0.9 or group-specific pharmacodynamic response. Future studies should therefore assess sugammadex response with detailed quantitative neuromuscular monitoring in sarcopenic LT recipients.

A significantly longer ICU length of stay was observed in the sarcopenic group, corresponding to a 50% longer median ICU stay. This difference likely reflects prolonged mechanical ventilation requirements, delayed extubation, increased pulmonary complications, infectious burden, and slower overall recovery. In the secondary model, each additional ICU day was associated with an approximately 9% increase in mortality risk. However, ICU length of stay should be interpreted primarily as a marker of postoperative disease severity and clinical trajectory rather than as an independent baseline predictor of mortality. Previous studies have similarly reported that prolonged ICU stay is associated with increased mortality risk and adverse postoperative outcomes, including delirium, renal failure, and long-term cognitive impairment [59,60].

The higher mortality observed in the sarcopenic group (27.1% vs. 8.7%) is consistent with previous studies and the 2024 meta-analysis by Prokopidis et al. [6]. In the primary model of the present study, sarcopenia was associated with an approximately threefold higher mortality risk; however, this association should be interpreted cautiously given the observational design and the possibility of residual confounding related to baseline disease severity, age, anemia, and perioperative complexity. Lower hemoglobin was also associated with increased mortality risk, indicating the clinical relevance of anemia in this high-risk population. Additionally, the increased intraoperative erythrocyte transfusion requirement in sarcopenic patients may reflect greater surgical complexity and impaired physiological reserve, rather than being solely attributed to structural factors such as vascular fragility [61]. The absence of a significant difference in total hospital length of stay between groups may be explained by post-ICU determinants, including graft function, immunosuppressive management, and standardized discharge protocols, which may attenuate early postoperative differences.

These outcome patterns also support the broader concept that conventional liver disease severity scores may not fully capture physiological reserve in LT candidates. Consistent with these findings, multiple studies have demonstrated that sarcopenia has been reported as an independent prognostic marker of disease severity and mortality in ESLD and that incorporating sarcopenia into the MELD score improves its prognostic accuracy [62,63]. Evidence supporting composite models such as MELD-sarcopenia over MELD or Child–Pugh scores alone is increasing [62,63]. In the present study, the higher MELD score observed in the sarcopenic group, together with the increased ICU length of stay and mortality, further supports the clinical relevance of this integrated approach. Therefore, risk stratification based solely on MELD or Child–Pugh scores may be insufficient in LT candidates. Incorporating sarcopenia into LT candidate assessment may provide a more comprehensive evaluation of physiological reserve and help optimize perioperative management, including individualized dosing, neuromuscular monitoring strategies, timely reversal strategies, and postoperative ICU resource planning.

### Limitations

This study has several limitations. (i) The single-center observational design limits the generalizability of the findings and precludes definitive causal inferences. In addition, the complex pathophysiological interplay between sarcopenia and ESLD makes it difficult to fully disentangle the independent effects of sarcopenia from liver-related pharmacokinetic alterations. Therefore, the observed prolongation of T0 cannot be attributed exclusively to sarcopenia. (ii) The exclusion of LT recipients with inconclusive sarcopenia classification represents a potential source of selection bias. Although this criterion was applied to reduce misclassification of sarcopenia status, it may have increased the contrast between the sarcopenia and non-sarcopenia groups and limited the generalizability of the findings to borderline or clinically ambiguous cases. Future prospective studies should consider evaluating these borderline patients as a separate subgroup to better define their pharmacodynamic profile and clinical outcomes. (iii) Due to collinearity and conceptual overlap among muscle-related and biochemical variables, only representative parameters were included in the multivariable models. Sarcopenia was incorporated as a composite variable rather than including its individual components (e.g., PMI and handgrip strength) separately, to avoid duplication of closely related variables. Similarly, total bilirubin was selected as a single representative parameter, as it encompasses both direct and indirect fractions. This approach was applied to minimize collinearity and prevent model overfitting. (iv) ICU length of stay was analyzed in a separate secondary model rather than in the primary model, as it reflects the postoperative clinical course rather than baseline patient characteristics. Therefore, ICU length of stay should be interpreted as a marker of postoperative disease severity and clinical trajectory, rather than as an independent baseline predictor of mortality. (v) Additional dosing requirements after T3 recovery and total rocuronium consumption were not analyzed, which may have provided further insight into intraoperative pharmacodynamic variability and drug utilization patterns. In addition, because fewer patients reached later intraoperative recovery/redosing time points, particularly T3, the phase-specific T2/T3 analyses were based on smaller subgroups and should therefore be interpreted as exploratory. (vi) Although age was independently associated with sarcopenia, residual confounding related to age cannot be completely excluded. Future studies with age-matched cohorts are needed to better distinguish the effects of sarcopenia from those of biological aging. (vii) This study was also limited to rocuronium; conducting similar pharmacodynamic and pharmacokinetic evaluations for other non-depolarizing neuromuscular blocking agents, such as vecuronium and cisatracurium, may further improve the generalizability of these findings. Future multicenter prospective studies are needed to validate these results and to better define sarcopenia-guided individualized anesthetic strategies.

## 5. Conclusions

The present study found that sarcopenia was associated with altered rocuronium pharmacodynamics in LT recipients, primarily characterized by delayed onset of complete neuromuscular blockade. Age was independently associated with sarcopenic status, supporting the close relationship between sarcopenia and aging in this cohort. However, sarcopenia remained associated with prolonged T0 after adjustment for age and other baseline clinical variables, suggesting that the delayed onset observed in sarcopenic recipients was not explained solely by chronological age. Sarcopenic recipients also showed prolonged extubation time, longer ICU stay, and higher postoperative mortality. In the adjusted mortality model, sarcopenia remained independently associated with mortality, while lower hemoglobin levels were also associated with increased risk. These findings support the incorporation of sarcopenia into perioperative risk assessment and emphasize the importance of quantitative neuromuscular monitoring, individualized rocuronium titration, and careful airway management in sarcopenic LT recipients. However, given the observational design and exploratory nature of secondary neuromuscular recovery analyses, these findings should be validated in prospective multicenter studies.

## Figures and Tables

**Table 1:** Baseline demographic, clinical, sarcopenia-related, and biochemical characteristics according to sarcopenia status.

Variables	Non-Sarcopenia Group (*n* = 69)	Sarcopenia Group (*n* = 70)	Effect Estimate (95%CI)	*p*
Demographic and anthropometric variables				
Age (years)	47 (40–55)	54.0 (45–62)	+7.0 (+1.8 to +12.2)	0.025
Sex, male, *n* (%)	47 (68.1)	52 (74.3)	1.35 (0.65 to 2.83)	0.538
Height (cm)	170 (165–177)	172 (165–180)	+2.0 (−3.2 to +7.2)	0.268
Weight (kg)	78 (68–86)	68 (60–82)	−10.0 (−17.3 to −2.7)	0.031
IBW (kg)	66 (55–72)	68 (60–75)	+2.0 (−3.6 to +7.6)	0.111
BMI (kg/m^2^)	26.7 (22.2–29.4)	23.8 (20.6–28.0)	−2.9 (−5.3 to −0.5)	0.041
Liver disease severity and sarcopenia-defining variables				
MELD score	16 (13–20)	18 (15–23)	+2.0 (−0.4 to +4.4)	0.045
Child–Pugh score	8 (6–9)	8 (7–10)	0.0 (−1.5 to +1.5)	0.157
PMA (cm^2^)	18.5 (15.6–21.3)	9.0 (7.40–12.0)	−9.5 (−11.3 to −7.7)	<0.001
PMI (cm^2^/m^2^)	6.0 (5.7–7.0)	3.0 (2.4–3.9)	−3.02 (−3.49 to −2.55)	<0.001
Handgrip strength (kg)	40 (35–45)	13 (5–21)	−27.0 (−30.8 to −23.2)	<0.001
Laboratory variables				
ALT (IU/L)	45 (30–71)	46 (26–89)	0.0 (−18.1 to +18.1)	0.712
AST (IU/L)	64 (40–89)	73 (43–128)	+9.0 (−11.1 to +29.1)	0.259
Total bilirubin (mg/dL)	2.9 (1.6–5.5)	3.7 (2.0–7.2)	+0.81 (−0.74 to +2.36)	0.117
Direct bilirubin (mg/dL)	1.0 (0.4–2.3)	1.6 (0.7–3.3)	+0.64 (−0.17 to +1.45)	0.072
INR	1.3 (1.1–1.6)	1.3 (1.2–1.7)	0.00 (−0.17 to +0.17)	0.525
Albumin (g/dL)	2.9 (2.4–3.5)	2.8 (2.0–3.5)	−0.10 (−0.48 to +0.28)	0.339
Creatinine (mg/dL)	0.9 (0.7–1.1)	1.0 (0.7–1.2)	+0.10 (−0.07 to +0.27)	0.199
BUN	14.5 (11.2–19.6)	15 (12.6–22)	+0.67 (−2.34 to +3.68)	0.148
Hemoglobin (g/dL)	11.2 (9.8–13.2)	9.9 (8.9–12.2)	−1.30 (−2.48 to −0.12)	0.021
Platelets	119 (73–170)	119 (77–185)	0.0 (−33.3 to +33.3)	0.628

Data are presented as median (Q1–Q3) or number (%). Effect estimates for continuous variables are presented as median differences with 95% confidence intervals, calculated as sarcopenic minus non-sarcopenic. For sex, the effect estimate is presented as the crude odds ratio for male sex in the sarcopenic group compared with the non-sarcopenic group. For continuous variables, *p*-values were derived from Mann–Whitney U tests, whereas median differences and their 95% confidence intervals were estimated using median quantile regression at q = 0.50. ALT: alanine aminotransferase; AST: aspartate aminotransferase; BUN: blood urea nitrogen; IBW: ideal body weight; BMI: body mass index; PMA: psoas muscle area; PMI: psoas muscle index; MELD: Model for End-Stage Liver Disease; INR: international normalized ratio.

**Table 2:** Intraoperative neuromuscular blockade kinetics, recovery/redosing time points (T0–T3), phase-specific patterns, and surgical characteristics according to sarcopenia status.

Variables	Non-Sarcopenia Group (*n* = 69)	Sarcopenia Group (*n* = 70)	Effect Estimate (95%CI)	*p*
T0—Time to TOF zero (second)	80 (70–98)	100 (76–135)	+20.0 (+4.0 to +36.0)	<0.001
Time to first recovery sign (T1) (min)	165 (94–222)	192 (132–285)	+27.0 (−24.8 to +78.8)	0.016
Time to second recovery sign (T2) (min)	263 (179–438)	330 (250–434)	+74.0 (−34.2 to +182.2)	0.272
Time to third recovery sign (T3) (min)	345 (237–497)	420 (327–560)	+90.0 (−38.7 to +218.7)	0.036
T1, dissection phase, (*n*; %)	51/57 (89.5)	25/36 (69.4)	0.27 (0.09 to 0.81)	0.031
T1, neohepatic phase, (*n*; %)	3/57 (5.3)	10/36 (27.8)	6.92 (1.75 to 27.31)	0.004
T2, dissection phase, (*n*; %)	23/48 (47.9)	13/28 (46.4)	0.94 (0.37 to 2.40)	1.000
T2, neohepatic phase, (*n*; %)	21/48 (43.8)	13/28 (46.4)	1.11 (0.44 to 2.84)	1.000
T3, dissection phase, (*n*; %)	11/31 (35.5)	1/17 (5.9)	0.11 (0.01 to 0.98)	0.035
T3, neohepatic phase, (*n*; %)	18/31 (58.1)	14/17 (82.4)	3.37 (0.80 to 14.18)	0.165
Duration of surgery (hours)	10 (9–11)	11 (9–12)	+0.5 (−0.2 to +1.2)	0.040
Intubation score (5–20 points)	5 (5–6)	5 (5–5)	0.0 (−0.3 to +0.3)	0.037
ES transfusion (units)	2 (1–4)	4 (2–5)	+2.0 (+1.2 to +2.8)	0.054

Data are presented as median (Q1–Q3) or number (%). Effect estimates for continuous variables are presented as median differences with 95% confidence intervals, calculated as sarcopenic minus non-sarcopenic. Effect estimates for phase-specific categorical variables are presented as crude odds ratios with 95% confidence intervals for the sarcopenic group compared with the non-sarcopenic group. For continuous variables, *p*-values were derived from Mann–Whitney U tests, whereas median differences and their 95% confidence intervals were estimated using median quantile regression at q = 0.50. TOF: train-of-four; T0: time to complete neuromuscular block; T1, T2, and T3: first, second, and third intraoperative neuromuscular recovery/redosing time points, respectively; ES: erythrocyte suspension.

**Table 3:** Postoperative outcomes according to sarcopenia status.

Variables	Non-Sarcopenia Group (*n* = 69)	Sarcopenia Group (*n* = 70)	Effect Estimate (95%CI)	*p*
ICU length of stay (days)	6.0 (4.0–8.0)	9.0 (6.0–17.0)	+3.0 (+0.6 to +5.4)	<0.001
Hospital length of stay (days)	30 (21–40)	29 (21–42)	−1.0 (−8.3 to +6.3)	0.831
Time to extubation (hours)	7 (5–9)	10 (8–14)	+3.0 (+1.5 to +4.5)	<0.001
Mortality (*n*; %)	6 (8.7)	19 (27.1)	3.91 (1.45 to 10.52)	0.009

Data are presented as median (Q1–Q3) or number (%). Effect estimates for continuous outcomes are presented as median differences with 95% confidence intervals, calculated as sarcopenic minus non-sarcopenic. For continuous variables, *p*-values were derived from Mann–Whitney U tests, whereas median differences and their 95% confidence intervals were estimated using median quantile regression at q = 0.50. Mortality is presented as the crude odds ratio with 95% confidence interval for the sarcopenic group compared with the non-sarcopenic group. ICU: intensive care unit.

**Table 4:** Comparison of baseline, intraoperative, and postoperative variables between survivors and non-survivors.

Variables	Survivors (*n* = 114)	Non-Survivors (*n* = 25)	Effect Estimate (95%CI)	*p*
Age (years)	51 (42–59)	55 (47–64)	+4.0 (−3.7 to +11.7)	0.067
Sex, male, *n* (%)	82 (71.9)	17 (68.0)	0.83 (0.33 to 2.11)	0.881
IBW (kg)	66.5 (59.0–73.0)	66.0 (56.0–72.0)	−1.0 (−7.8 to +5.8)	0.548
BMI (kg/m^2^)	24.2 (21.2–28.7)	27.7 (22.5–36.3)	+3.5 (+0.02 to +7.0)	0.065
MELD score	16.5 (14.0–20.0)	17.0 (15.0–24.0)	+1.0 (−1.7 to +3.7)	0.267
Child–Pugh score	8.0 (7.0–9.0)	8.0 (6.0–10.0)	0.0 (−1.9 to +1.9)	0.894
PMA (cm^2^)	14.1 (9.2–19.0)	9.40 (6.5–15.6)	−4.7 (−8.8 to −0.6)	0.004
PMI (cm^2^/m^2^)	5.0 (3.2–6.1)	3.2 (2.5–5.2)	−1.77 (−3.06 to −0.48)	0.003
Handgrip strength (kg)	32 (15–42)	15 (5–26)	−17.0 (−27.4 to −6.6)	0.009
ALT (IU/L)	46 (30–80)	35 (19–88)	−10.0 (−34.9 to +14.9)	0.310
AST (IU/L)	68 (40–104)	60 (43–114)	−8.0 (−35.6 to +19.6)	0.659
Total bilirubin (mg/dL)	3.0 (1.6–5.9)	4.3 (2.7–9.2)	+1.35 (−0.69 to +3.39)	0.014
Direct bilirubin (mg/dL)	1.1 (0.5–2.8)	2.1 (1.0–4.9)	+1.02 (−0.06 to +2.10)	0.031
INR	1.3 (1.1–1.6)	1.40 (1.2–1.7)	+0.10 (−0.13 to +0.33)	0.173
Albumin (g/dL)	2.9 (2.4–3.5)	2.9 (2.0–3.2)	0.00 (−0.50 to +0.50)	0.459
Creatinine (mg/dL)	0.9 (0.7–1.1)	0.9 (0.7–1.1)	0.00 (−0.18 to +0.18)	0.796
BUN	14.3 (11.7–21.0)	15.9 (13.6–23.0)	+1.69 (−2.53 to +5.91)	0.188
Hemoglobin (g/dL)	10.9 (9.6–13.2)	9.0 (8.4–11.5)	−1.90 (−3.53 to −0.27)	0.003
Platelets	114 (75–178)	143 (76–169)	+33.0 (−11.8 to +77.8)	0.389
Duration of surgery (hours)	10.2 (9.3–11.2)	10.3 (9.3–11.5)	+0.1 (−0.8 to +1.0)	0.395
Intubation score (5–20 points)	5 (5–6)	5 (5–6)	0.0 (−0.5 to +0.5)	0.745
T0	90 (70–115)	92 (70–150)	+3.0 (−17.8 to +23.8)	0.184
T1	172 (103–230)	206.5 (162–255)	+51.0 (−18.4 to +120.4)	0.096
T2	285 (182–435)	340 (285–432)	+60.0 (−91.0 to +211.0)	0.291
T3	375 (253–460)	495 (435–527)	+135.0 (−44.5 to +314.5)	0.014
ES transfusion (units)	3 (1–5)	4 (2–6)	+1.0 (−0.9 to +2.9)	0.019
ICU length of stay (days)	6.5 (5.0–10.0)	14.0 (8.0–22.0)	+8.0 (+4.8 to +11.2)	<0.001
Hospital length of stay (days)	30 (23–42)	19 (9–37)	−11.0 (−20.5 to −1.5)	0.002
Time to extubation (hours)	8 (6–12)	8.5 (8–9)	0.0 (−8.5 to +8.5)	0.872

Effect estimates for continuous variables are presented as median differences with 95% confidence intervals, calculated as non-survivors minus survivors. For continuous variables, *p*-values were derived from Mann–Whitney U tests, whereas median differences and their 95% confidence intervals were estimated using median quantile regression at q = 0.50. Male sex is presented as the crude odds ratio with 95% confidence interval for non-survivors compared with survivors.

**Table 5:** Multivariable logistic regression analysis of factors associated with mortality.

Variable	B	SE	Exp(B)	*p*-Value	95% CI
Primary model					
Sarcopenia	1.182	0.520	3.26	0.023	1.18–9.02
Hemoglobin	−0.222	0.111	0.80	0.046	0.64–0.99
Total bilirubin	0.046	0.043	1.06	0.213	0.97–1.15
Secondary model					
ICU length of stay (per day)	0.089	0.023	1.09	<0.001	1.05–1.14

Due to collinearity and conceptual overlap, only one representative variable from correlated parameters was included in the multivariable model. Specifically, sarcopenia was selected to represent muscle-related parameters, while total bilirubin was included instead of direct bilirubin, as it encompasses both direct and indirect fractions.

## Data Availability

The data presented in this study are available on request from the corresponding author. The data are not publicly available due to privacy and ethical restrictions.
